# Nicotinamide is an endogenous agonist for a *C. elegans* TRPV OSM-9 and OCR-4 channel

**DOI:** 10.1038/ncomms13135

**Published:** 2016-10-12

**Authors:** Awani Upadhyay, Aditya Pisupati, Timothy Jegla, Matt Crook, Keith J. Mickolajczyk, Matthew Shorey, Laura E. Rohan, Katherine A. Billings, Melissa M. Rolls, William O. Hancock, Wendy Hanna-Rose

**Affiliations:** 1 Department of Biochemistry and Molecular Biology, The Pennsylvania State University, University Park, Pennsylvania 16802, USA; 2 Molecular Cellular and Integrative Biosciences Graduate Program, The Pennsylvania State University, University Park, Pennsylvania 16802, USA; 3 Department of Biology, The Pennsylvania State University, University Park, Pennsylvania 16802, USA; 4 Department of Biomedical Engineering, The Pennsylvania State University, University Park, Pennsylvania 16802, USA; 5 Interdisciplinary Graduate Degree Program in Bioengineering, The Pennsylvania State University, University Park, Pennsylvania 16802, USA

## Abstract

TRPV ion channels are directly activated by sensory stimuli and participate in thermo-, mechano- and chemo-sensation. They are also hypothesized to respond to endogenous agonists that would modulate sensory responses. Here, we show that the nicotinamide (NAM) form of vitamin B_3_ is an agonist of a *Caenorhabditis elegans* TRPV channel. Using heterologous expression in Xenopus oocytes, we demonstrate that NAM is a soluble agonist for a channel consisting of the well-studied OSM-9 TRPV subunit and relatively uncharacterized OCR-4 TRPV subunit as well as the orthologous *Drosophila* Nan-Iav TRPV channel, and we examine stoichiometry of subunit assembly. Finally, we show that behaviours mediated by these *C. elegans* and *Drosophila* channels are responsive to NAM, suggesting conservation of activity of this soluble endogenous metabolite on TRPV activity. Our results in combination with the role of NAM in NAD+ metabolism suggest an intriguing link between metabolic regulation and TRPV channel activity.

## Abstract (web summary)

TRPV are cation channels activated by physical and chemical stimuli. Here the authors show that nicotinamide is a soluble, endogenous agonist for orthologous TRPV channels from *C*. *elegans* and *Drosophila*, unveiling a metabolic-based regulation for TRPV channel activity.

## Graphical abstract

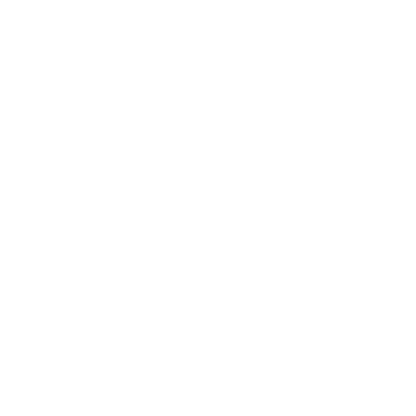

TRPV channels are non-specific cation channels that are expressed in specialized sensory neurons as well as some non-sensory cells^1^,^2^,^3^. They mediate behavioural responses to exogenous chemical, mechanical and temperature stimuli^3^,^4^,^5^. However, endogenous modulators of TRPV channel activity are also important for regulation of behavioural responses^6^,^7^, and identification of cellular metabolites that impact channel activity is of interest for revealing potential links between sensory function and metabolic state.

TRPV family proteins assemble as homomeric or heteromeric tetramers, with subunit composition determining functional specificity^8^,^9^,^10^,^11^,^12^. *osm-9* is the most widely-expressed TRPV gene in *Caenorhabditis elegans* and has the most attributable functions, including roles in mechano- and odorant sensation as well as behavioural adaptation responses^13^,^14^. Other *C. elegans* TRPV family genes called *ocr* genes (*osm-9* and capsaicin receptor related) are expressed in subsets of *osm-9*-expressing cells, and OCR subunits are hypothesized to function in heteromeric combination with OSM-9 (refs 5, 14, 15). OSM-9 and OCR-2 share the most functions^13^,^14^,^16^,^17^, and the two proteins are mutually required to achieve localization to the ciliated endings in the ASH cells, consistent with function as subunits of the same heteromeric channel^14^. However, some *osm-9* functions are *ocr-2* independent and *vice versa*^14^,^18^. Multiple attempts to demonstrate OCR-2 and OSM-9 homomeric or heteromeric channel activity in a heterologous expression system have not been successful^13^,^14^,^18^, leading to speculation that other unknown subunits are required for assembly of an active channel^14^,^19^.

While expression patterns of *ocr-1*, *ocr-3* and *ocr-4* are described^14^, these genes are less well characterized. *ocr-1, ocr-2* and *ocr-4* have redundant function in four neuroendocrine cells called the uterine vulval one (uv1) cells where they act to inhibit egg laying^18^. The uv1 cells are thought to function by responding to uterine stretching to relieve this inhibition^18^,^20^. *osm-9* is expressed in uv1 cells, but is surprisingly not involved in egg laying^14^,^18^. Thus, heteromeric combinations of unknown composition are proposed to function in uv1 cells^18^. Here, we demonstrate that OSM-9 and OCR-4 form a functional channel in the uv1 cells.

Nicotinamide (NAM) is a form of vitamin B_3_ that is absorbed from the diet and is derived endogenously from hydrolysis of NAD^+^ by NAD^+^ consumer enzymes such as sirtuins and PARPs. Cells use NAM to resynthesize NAD^+^, and in invertebrates the nicotinamidase PNC-1 catalyses the first step in this biosynthetic NAD^+^ salvage pathway (Fig. 1a). In the absence of PNC-1 activity in *C. elegans*, NAM levels are increased as much as tenfold^21^. It was reported that uv1 cells undergo a NAM-dependent death in *pnc-1* mutants^22^,^23^.

Here, we report an additional NAM-dependent death within the nervous system and the molecular mechanism by which NAM triggers cell death. We show that the genes *osm-9* and *ocr-4* are required for NAM-induced cell deaths, and we use electrophysiology to reveal that NAM, an endogenous metabolite, acts as an evolutionarily conserved agonist for the TRPV channel encoded by *osm-9* and *ocr-4* in *C. elegans* and by *iav* and *nan* in *Drosophila*. We also show that NAM modulates behavioural responses in both *C. elegans* and *Drosophila* by acting as a TRPV channel agonist.

## Results

### Two cell-types die in *pnc-1* mutants

**Figure 1:**
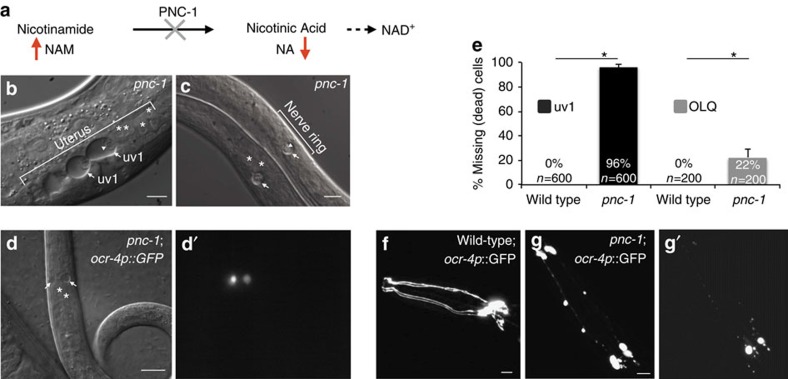
NAM-induced cell death affects two cell types in *pnc-1* mutants. (**a**) PNC-1 converts NAM to NA during salvage biosynthesis of NAD^+^. Loss of PNC-1 results in accumulation of NAM. Both the **b** uterine uv1 cells and the **c** dying cells in the nerve ring in *pnc-1(pk9605)* null mutants (strain HV560) have a similar necrotic morphology. The cell bodies swell to many times normal size (arrows), while the nuclear membrane disintegrates and the nucleus moves to the cell periphery (arrowheads). (**d**) DIC image and **d**′ corresponding fluorescence image of a *pnc-1* mutant carrying the *psEx279*[*ocr-4*p::GFP] transgene. The dying cells in the region of the nerve ring (arrows) are marked by GFP. For comparison to swollen cells, multiple cells of normal morphology and size are annotated in **b**–**d** with an asterisk directly above the nucleus and in Supplementary Fig. 1. (**e**) Quantification of the cell deaths in *pnc-1* null mutants. uv1 cell death (black bars) was scored in late L4 according to the absence of cells with the *inIs179*[*ida-1*::GFP] marker (strain HV560), and OLQ (grey bars) death was scored in late L3 or L4 according to the absence of cells with the *psEx279*[*ocr-4p*::GFP] marker (strain HV695). Actual per cent missing cells and sample size (number of cells examined) is indicated on each bar. Error bars are 99% confidence intervals (not applicable to 0%). *P<0.0001, calculated using Fisher's exact test. (**f**) The dendrites of the OLQ cell extend to the tip of the nose, visualized in a wild-type animal carrying the *ocr-4p*::GFP transgene. (**g**,**g**′) Fluorescence images of the (**g**) late L3 and (**g**′) L4 stage of the same *pnc-1; psEx279*[*ocr-4p*::GFP] animal, demonstrating that death is a degenerative process involving (**g**) first the blebbing of the dendrites (**g**′) followed by their disappearance as the cell bodies round up and begin to disappear. Note that the image in **g**′ is overexposed relative to **g**. Scale bars in all panels are 10 μm.

uv1 cell death is highly conspicuous in *pnc-1* mutants because of the excessive size of the dying cells (Fig. 1b), the almost complete penetrance of the phenotype (Fig. 1e), and the persistence of the swollen cell corpses into early adulthood^23^. However, we speculated that one or more neurons were also dying with incomplete penetrance in the *pnc-1* mutant because we observed the occasional presence of vacuoles resembling the swollen uv1 corpses in the region of the nerve ring of some young larvae (Fig. 1c). By using a panel of GFP markers to visualize specific neurons, we found that *ocr-4p::gfp (psEx279),* a marker of OLQ cell fate^18^, consistently marked the swollen cells (Fig. 1d). Moreover, the absence of GFP in older animals revealed that 22% of OLQ cells are missing by late larval stages in *pnc-1* mutants (Fig. 1e). We conclude that the observed vacuoles are swollen, dying OLQ cells and that no other head neurons are affected morphologically by loss of *pnc-1* because there are no corpses that lack the GFP label in the *ocr-4p*::*gfp*; *pnc-1* animals.

The morphology of the dying OLQ neurons resembles that of the necrotic uv1 cells^24^; the cell body swells (Fig. 1b,c, arrows) and the nuclear membrane disintegrates while the nucleus moves to the periphery of the cell (Fig. 1b,c, arrowheads). The OLQ dendrites first bleb (Fig. 1g) and then appear fragmented by the time the OLQ cell body appears swollen (Fig. 1ǵ). Both the cell body and the neurites disappear by adulthood, although GFP positive remnants of the corpse can be seen in the head infrequently. Like uv1 cell necrosis, OLQ death is not dependent on apoptotic genes *ced-3* or *ced-4* (Supplementary Fig. 2)^23^. We conclude that OLQ cells undergo a necrotic death similar to that of uv1 cells in the *pnc-1* mutant.

### Acute NAM treatment causes OLQ and uv1 cell death

**Figure 2:**
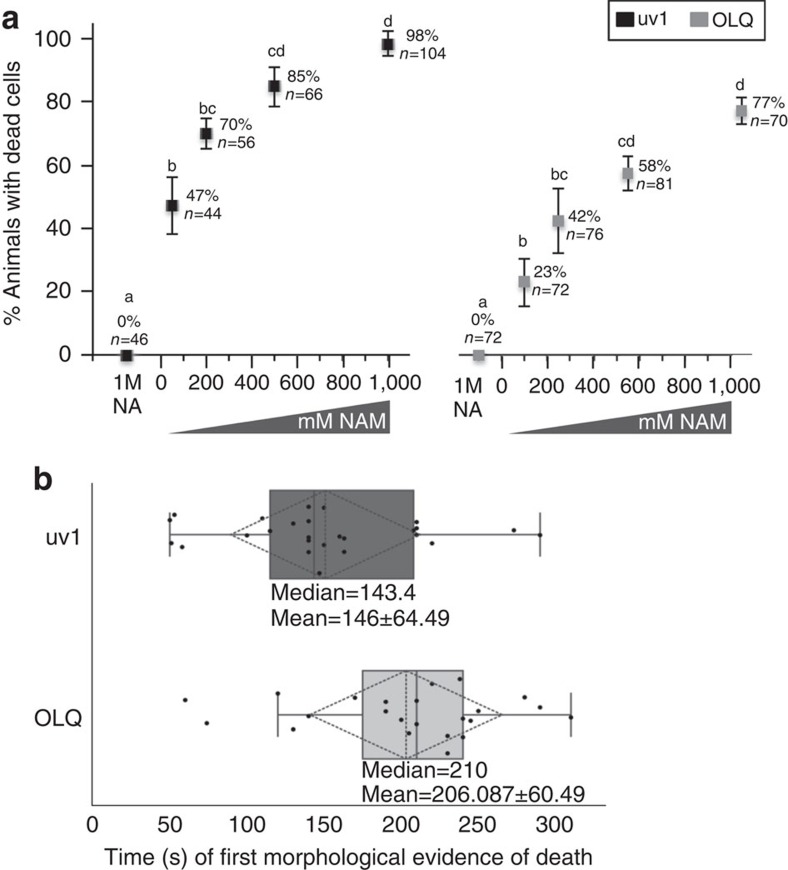
Acute application of NAM results in rapid death of uv1 and OLQ cells. (**a**) 50, 200, 500 or 1,000 mM NAM or 1,000 mM NA (control) was added to individual animals and the percentage of animals with dying uv1 (black markers) or OLQ cells (grey markers) was quantified. Actual percentages and total number of animals examined (*n*) are indicated for each data point. Values represent the average from at least three supplementation experiments and error bars are standard deviations. For statistical analysis, *P* values for all pairwise comparisons were calculated using Fisher's exact test with a Bonferroni correction considering 10 total pair-wise comparisons for each experiment. Data points marked with distinct annotations (a–d) within each experiment indicate statistical differences with a Bonferroni adjusted *P*<0.001. (**b**) Cell death in response to acute NAM treatment is rapid. Each dot represents an individual animal and the position on the *X*-axis represents the time after NAM addition when the first evidence of cell death, nuclear disintegration, was evident. Boxes show the upper and lower quartile values, solid and dashed vertical lines indicate the median and mean of the population distribution, respectively. The dotted rhombus indicates one standard deviation, and error bars indicate the maximum and minimum of the population distribution. Two of the data points for OLQ were statistical outliers and not included in the statistical analysis.

uv1 necrosis in *pnc-1* mutants can be recapitulated in wild-type animals by supplementation of cultures with NAM (ref. 22). Thus, we tested the effect of acute application of NAM to wild-type animals. Application of a NAM solution to animals causes death of uv1 and OLQ cells in a dose-dependent manner (Fig. 2a). Acute application of NAM kills uv1 cells more effectively than OLQ cells (Fig. 2a), and the highest concentrations of NAM result in more penetrant death of OLQ cells (Fig. 2a) relative to genetic removal of *pnc-1* (Fig. 1e). NAM-induced death is not a response to a change in osmolarity and is specific to the amide form of vitamin B_3_; 1 M nicotinic acid (NA) has no effect on uv1 or OLQ cells (Fig. 2a). The NAM-induced necrotic process is remarkably rapid, advancing to the first morphological signs of death, nuclear membrane disintegration, within 50 s of NAM application in some animals (Fig. 2b). The OLQ cell response is somewhat delayed on average in addition to being less penetrant relative to uv1 cells (Fig. 2b).

### NAM-induced death requires *ocr-4* and *osm-9* gene function

**Figure 3:**
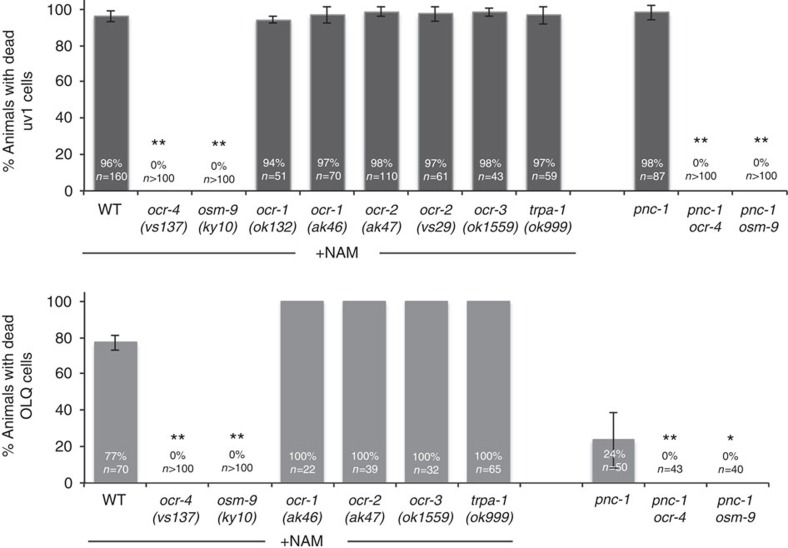
An OCR-4–OSM-9 TRPV channel mediates NAM-induced cell death. The percentage of animals with dead uv1 cells (black bars) or OLQ cells (grey bars) for each genotype supplemented with 25 mM NAM (+NAM) or in combination with *pnc-1(pk9605)* is reported. Actual percentages and sample sizes (*n*=number of animals examined) are indicated on each bar. Reported values for NAM supplemented animals are the average of at least three experiments and error bars are standard deviation. Error bars for the *pnc-1* double mutant strains represent 95% confidence intervals of the proportion of the population (not applicable to values of 0 or 100%.) The wild-type strain used is N2. All alleles are loss or reduction of function with the exception of *ocr-2(vs29)* which is a dominant negative allele^18^. For statistical analysis, *P* values for all pairwise comparisons were calculated using Fisher's exact test followed by a Bonferroni correction considering eight (+NAM) or two (*pnc-1*) total pair-wise comparisons. ** Bonferroni adjusted *P*<0.001. * Bonferroni adjusted *P*=0.0018.

The *ocr-4p::gfp* transgenic animals, which we used to visualize OLQ cells, have visible GFP expression in one other cell type, the uv1 cells^18^. This provocative expression pattern of the reporter for the TRPV channel subunit OCR-4 exclusively in the two dying cell types led us to investigate the functional relationship between OCR-4 and NAM-induced death. We found that mutation of *ocr-4* completely prevents death of both uv1 and OLQ cells caused by either loss of *pnc-1* or acute application of NAM (Fig. 3). *osm-9*, *ocr-1* and *ocr-2* are co-expressed with *ocr-4* in uv1 cells, and *osm-9* is co-expressed with *ocr-4* in OLQ (refs 14, 18). Thus, we predicted that OSM-9 might be required as the heteromeric partner for OCR-4 in NAM-induced necrosis. Indeed, mutation of *osm-9* also completely prevents NAM-induced death (Fig. 3). We also examined the other *ocr* genes and a related TRPA1 gene, *trpa-1,* for a role in mediating NAM-induced death. However, mutation of these genes had no effect on death of either cell type (Fig. 3).

### NAM is an agonist for an OSM-9 and OCR-4 channel

**Figure 4:**
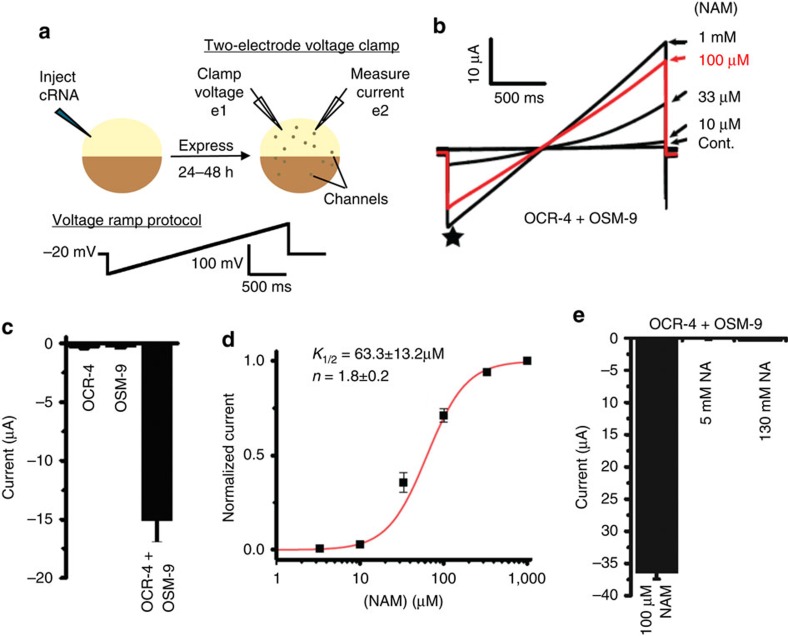
NAM activates the OCR-4 and OSM-9 heteromeric TRPV channel. (**a**) Diagram of 2-electrode voltage clamp experiment and voltage ramp protocol: Step 1: cRNA is injected in the *Xenopus* oocyte. In 1–2 days, ion channel proteins express on the oocyte membrane. Step 2: Oocytes are impaled with two microelectrodes; e1 and e2. Command voltage (Vc) is chosen and kept constant using a feedback amplifier. Any change in oocyte membrane potential (Vm) due to ion channel activity is sensed by e1. To make Vm=Vc, a current is injected in the oocyte by e2. The injected current, which is equal and opposite to the current generated by ion channel activity, is measured. Currents were recorded in response to 2 s voltage ramps from −100 to +100 mV from a −20 mV hold. (**b**–**d**) Large NAM-activated currents were observed in oocytes co-expressing OCR-4 or OSM-9, but not expressing each subunit individually. Current size was measured at −100 mV (star), and the half maximal concentration (*K*_1/2_) for current activation by NAM was determined from the Hill plot shown in **d**. *n* indicates the Hill coefficient. The data in **d** show mean±s.e.m. (*n*=5–9 oocytes per NAM concentration), and the red curve is a Hill equation fit. Data from individual oocytes were normalized to the current amplitude observed at 1 mM NAM before comparison. (**e**) 5 mM NA did not elicit large currents in OSM-9–OCR-4-expressing oocytes (−0.154±0.018 μA, *n*=6 oocytes), but there is a slight increase in current upon exposure to 130 mM NA (−0.269±0.026 μA, *n*=4). Though this is a statistically significant increase (*P*<0.05, two-tailed *t*-test), 130 mM NA still elicited over 200-fold less current than 100 μM NAM (−36.5±1.0 μA, *n*=4).

The rapid execution of death upon acute NAM treatment and the striking morphology of the dying cells is reminiscent of excitatory death due to direct membrane depolarization caused by degenerin mutations in *C. elegans*^25^. Because OSM-9 channels mediate influx of divalent cations^26^, we hypothesized that NAM is an agonist for a channel containing OSM-9 and OCR-4 subunits and that excessive activation of the channel may lead to excitotoxicity. To test the hypothesis, we expressed OSM-9 and OCR-4 individually and in combination in *Xenopus* oocytes and used electrophysiology to determine whether the resulting channel could be activated by NAM (Fig. 4a). Indeed, NAM activates a large current selectively in oocytes co-expressing OCR-4 and OSM-9 (Fig. 4b,c and Supplementary Fig. 3). The *K*_1/2_ value for NAM, derived from a Hill fit of current size, is 63.3±13.2 μM (Fig. 4d). This value suggests that OSM-9–OCR-4 channel activation will be elevated in *pnc-1* mutants given the difference in NAM concentrations found in lysates from wild-type (15.7 μM) and *pnc-1* mutant (88 μM) animals^21^. The agonist activity of NAM is not shared by the related metabolite NA; 100 μM NAM was greater than 100-fold more potent than a 1,000-fold higher concentration of NA (Fig. 4e). We conclude that the NAM form of vitamin B_3_, but not the NA form, is a potent agonist for a TRPV channel consisting of OSM-9 and OCR-4.

### The OSM-9–OCR-4 channel likely has two of each subunit

**Figure 5:**
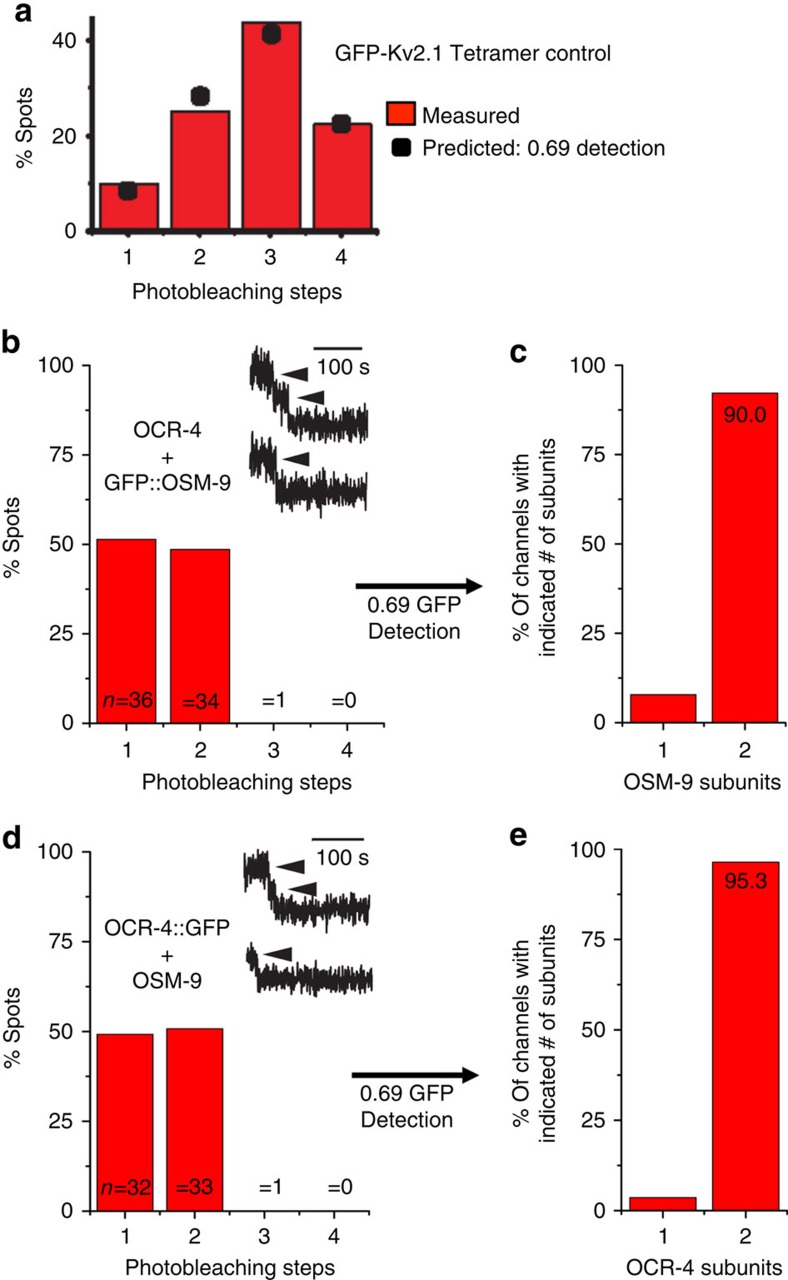
Stoichiometry of OSM-9–OCR-4 channels. (**a**) Frequency of the number of bleaching steps observed for 113 channels from oocytes expressing GFP-Kv2.1 homotetramers. The predicted step count distribution assuming 0.69 GFP detection probability (black symbols) is a best least squares fit of the data. (**b**) Frequency of the number of bleaching steps observed for 71 channels from oocytes expressing GFP::OSM-9–OCR-4. The inset shows example fluorescent traces for two-step (top) and one-step (below) photobleaching. (**c**) Stoichiometry distribution calculated from the observed one and two-step event totals in **b**, assuming a 0.69 GFP detection efficiency. (**d**) Frequency of the number of bleaching steps observed for 66 channels from oocytes expressing OSM-9–OCR-4::GFP. (**e**) Stoichiometry distribution calculated from one and two-step events in **d**, assuming a 0.69 GFP detection efficiency.

The ability to detect active channels provides an opportunity to examine the stoichiometry of subunits in the active heteromeric channel, an unanswered question regarding the *C. elegans* TRPV channels. To determine stoichiometry, we used an established total internal reflection (TIRF) microscopy photobleaching assay that involves counting the number of GFP-labelled subunits in channels that successfully traffic to the plasma membrane^27^,^28^. We first labelled subunits via fusion of GFP to the carboxy terminus of OCR-4 and to the amino terminus of OSM-9 and demonstrated that fusion to GFP did not interfere with assembly or function of channels. Expression of OCR-4::GFP with OSM-9 or GFP::OSM-9 with OCR-4 at 1:1 ribonucleic acid ratios each resulted in NAM-responsive channels (average currents of 18.50±2.56 μA, *n*=5 eggs, and 25.00±2.86 μA, *n*=6, in response to 100 μM NAM, respectively). As a control for our TIRF methodology, we first quantified the distribution of channels bleaching in one to four steps using a known homotetrameric potassium channel, Kv2.1, tagged with GFP (Fig. 5a). The assay faithfully reports a maximum of four detectable subunits in the Kv2.1 channel (Fig. 5a). On the basis of the distribution of channels bleaching in one to four steps, we calculate a GFP detection efficiency of 0.69 in our TIRF system, a value consistent with other published studies^27^,^29^,^30^.

Upon injection of OCR-4::GFP or GFP::OSM-9 alone, no validated channels were observed at the plasma membrane (for example, Supplementary Fig. 4b). However, when we expressed OCR-4::GFP with OSM-9 or GFP::OSM-9 with OCR-4 at 1:1 ribonucleic acid ratios, we observed many putative channels at the plasma membrane (for example, Supplementary Fig. 4a). We quantified the distribution of all channels that bleach in a predicted stepwise fashion and found that bleaching occurs in either one or two steps in each of the reciprocal experiments (Fig. 5b,d and Supplementary Fig. 4), suggesting that a maximum of two of each subunit insert into the heteromeric channels. Then, using the GFP detection efficiency value (0.69) determined with our control (Fig. 5a), we calculated that greater than 90% of channels adopt a 2:2 stoichiometry in each of the two independent reciprocal experiments (Fig. 5c,e). We did observe one spot that bleached in three steps for each experiment (Fig. 5b,d). Thus, we cannot rule out the formation of a small proportion of channels with 3:1 or 1:3 OSM-9:OCR-4 stoichiometry. However, the majority of stable membrane-localized channels observed in this functional expression experiment are comprised of two of each subunit (Fig. 5c,e).

### NAM-induced OLQ cell death causes a behavioural phenotype

**Figure 6:**
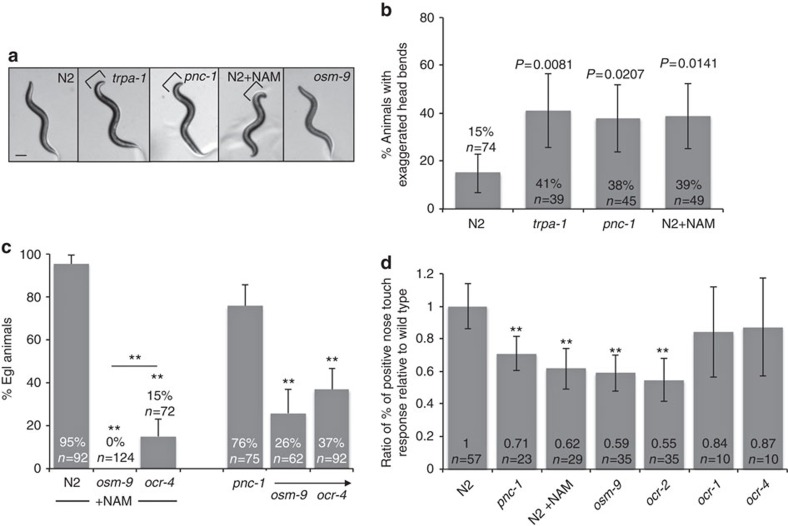
NAM induced behaviours are mediated by *osm-9* and *ocr-4* expressing cells. (**a**) Images of L4 animals documenting the foraging, exaggerated nose-bending phenotype (brackets). Scale bar is 100 μm. (**b**) *pnc-1* mutants and NAM supplemented animals have a foraging phenotype consistent with *trpa-1* mutants. Error bars are 95% confidence intervals. Actual percentages and number of animals examined is indicated in each bar. *P* values were calculated using Fisher's exact test with a Bonferroni correction considering three pair-wise comparisons. (**c**) The NAM-induced Egl phenotype depends on *osm-9* and *ocr-4* function. Error bars are 95% confidence intervals. Actual percentages and number of animals examined is indicated in each bar. **Bonferroni adjusted *P*<0.001. (**d**) *pnc-1* mutant and NAM-treated animals have a nose touch defect like that of *osm-9* mutants, but *ocr-4* mutants do not share this phenotype. We touched each animal ten times, calculated the percentage of touches that resulted in a positive response and reported the ratio of per cent positive responses relative to wild type. The ratio and the number of animals examined for each genotype or condition is indicated on each bar. Error bars are 95% confidence intervals. **Bonferroni adjusted *P*<0.001.

We next explored if the cell deaths in *pnc-1* mutants affect animal behaviour and if other NAM-induced phenotypic effects are mediated by *osm-9* and/ or *ocr-4*. The OLQ are sensory cells with ciliary endings exposed to the environment through the nose. They play a prominent role in regulating head movements during foraging in response to mechanical sensation of food, and this function requires the autonomous activity of a gene called *trpa-1* (refs 31, 32, 33). We used the *trpa-1* mutant as a control animal with a known functional deficit of OLQ cells and observed that 41% of *trpa-1* mutants display an exaggerated head bending phenotype compared with only 15% of wild-type control animals (Fig. 6a,b). We similarly examined *pnc-1* mutants and NAM-supplemented wild-type animals and observed that they also display exaggerated head bending during foraging, (Fig. 6a,b), similar to the *trpa-1* mutant animals. *osm-9* mutants do not display this phenotype (Fig. 6a). These results suggest that the exaggerated head bending in both NAM supplemented and *pnc-1* mutant animals is an indirect result of TRPV-mediated OLQ cell death.

### A NAM-induced Egl phenotype is mediated by *ocr-4* and *osm-9*

Loss of uv1 cells is expected to promote egg laying by decreasing the time eggs are retained in the uterus before expulsion^18^. However, the egg-retention phenotype expected upon loss of uv1 cells cannot be evaluated in *pnc-1* mutants or NAM-supplemented wild-type animals because both fail to lay eggs, resulting in the death of the hermaphrodite upon internal hatching of embryos^22^. This egg-laying defect (Egl phenotype) is not a result of the lack of the uv1 cells; animals genetically engineered to lack uv1 cells lay eggs normally^34^.

Nevertheless, because neither *osm-9* nor *ocr-4* mutants have an Egl phenotype, we were able to test if the NAM-induced Egl phenotype was a result of NAM action as an agonist on an OSM-9–OCR-4 channel by examining if *osm-9* or *ocr-4* were required for NAM to induce the Egl defect. Indeed, supplementation with NAM does not induce an Egl phenotype in the absence of *osm-9* and is significantly less effective in the absence of *ocr-4* (Fig. 6c). Similarly, mutation of *osm-9* or *ocr-4* reduces the penetrance of the Egl defect in *pnc-1* mutants as well (Fig. 6c). The effect of NAM on egg laying could conceivably be indirect, caused when the swollen uv1 cell corpses interfere with egg-laying structures or processes. This type of indirect effect is not ruled out but is demonstrably not the only effect of NAM because the Egl phenotype is evident in *ocr-4* mutants, which have no uv1 cell corpses (Fig. 3). We previously concluded that the Egl phenotype in *pnc-1* mutants is attributable to impaired sex muscle function because of impaired muscle responses to agonist^35^. Thus, we conclude that NAM may act on a channel containing OSM-9 and OCR-4 (or alternative) subunits to interfere with muscle activity required for egg-laying function. These results suggest that OSM-9 and OCR-4 might be acting in a location previously unrecognized to express *osm-9* or *ocr-4* and that activation of the channel may interfere with cell function in some locations without causing death.

### NAM-responsiveness of other channels

The OSM-9–OCR-2 channel mediates gentle nose touch mechanosensation^15^. Intriguingly, both *pnc-1* mutants and NAM-supplemented animals have a deficit in responding to gentle nose touch (Fig. 5d). This phenotype mimics that of *osm-9* and *ocr-2* mutants but, intriguingly, is not shared by *ocr-4* mutants (Fig. 5d), suggesting that NAM may act on a TRPV channel that does not include the OCR-4 subunit. We attempted to test the ability of NAM to activate an OSM-9–OCR-2 channel in the heterologous expression system, but we detected no currents above baseline in co-injected oocytes in the presence of 300–1 M NAM (*n*=5 injections). We also considered whether the nose-touch phenotype could be a result of death of OLQ cells. However, the detected phenotype is more penetrant than the 10% reduction reported to occur from ablation of OLQ cells^36^, especially considering the incomplete penetrance of the OLQ death (Fig. 3.). We conclude that excessive NAM accumulation induces behavioural deficits consistent with impaired function of cells other than OLQ, without causing death. It remains possible that the cells that are impaired are OLQ that escape death in addition to its partners in the nose-touch circuit and that this cell impairment is more detrimental to nose touch function than OLQ cell absence.

### The orthologous *Drosophila* channel is responsive to NAM

**Figure 7:**
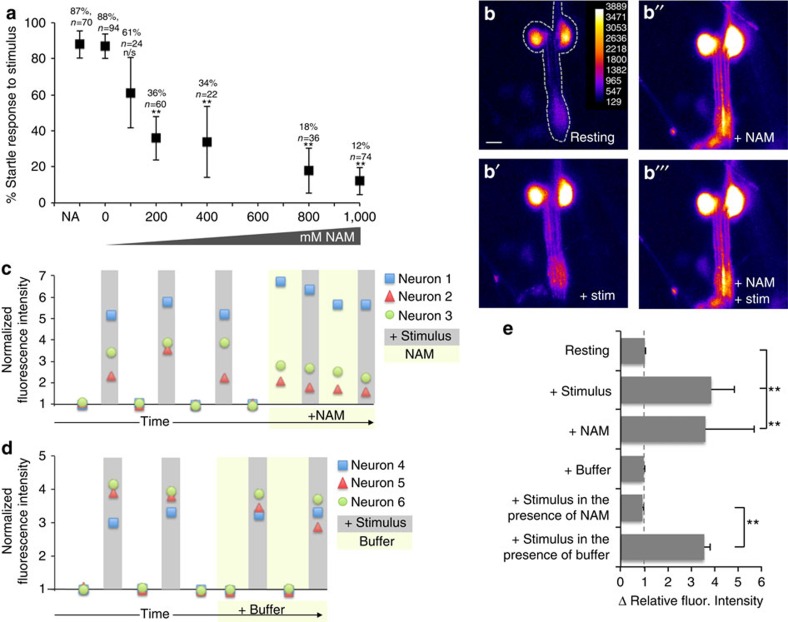
NAM affects *Drosophila* chordotonal neuron dependent phenotypes. (**a**) Third instar larva were pretreated with different concentrations of NAM or 1 M NA as a control for 10 min then exposed to a 500 Hz tone at 90 dB and scored according to presence or absence of a startle response. Data are presented as percentage of startle responses to the stimulus. Error bars are 95% confidence intervals. Actual percentages and sample sizes are indicated above each bar. For statistical analysis, *P* values for six pairwise comparisons to 0 mM NAM were calculated using Fisher's exact test followed by a Bonferroni correction. **Bonferroni adjusted *P*<0.001. (**b**) Fluorescence intensity of chordotonal neurons expressing GCaMP6 (**b**) increases in response to mechanical stimulus (**b**′), increases in response to application of 1 M NAM (**b**′′), but fails to further increase in response to stimulus after application of NAM (**b**′′′). Images are individual frames from Supplementary Movie 1. An example of the region of interest (ROI) used to quantify images is outlined in **b**. Scale bar is 10 μm. Fiji's ‘Fire' Heat map LUT (lookup table) has been applied to images for visualization of intensity differences. (**c**,**d**) Fluorescence intensity for each individual neuron examined is normalized to average baseline values and plotted at baseline, immediately after repeated applications of stimulus, after returning to base line, after application of NAM or buffer, and after subsequent reapplications of stimulus (spacing between stimuli is 16 s),. (**e**) Quantification of data from **c**,**d**. The change in normalized relative fluorescence intensity in response to stimulus, to application of buffer or NAM, and to a mechanical stimulus in the presence of NAM or buffer is plotted. Error bars are standard deviations. The dotted line marks no change. **P<0.001 calculated using a two-tailed *t* test with Bonferroni adjustment considering three pairwise tests.

To further assess if NAM responsiveness is a conserved feature of the invertebrate TRPV channel, we investigated the effect of NAM on the orthologous channel in *Drosophila*. OCR-4 and OSM-9 correspond to *Drosophila* Nanchung (Nan) and Inactive (Iav), respectively^3^,^37^. Nan and Iav form a heteromeric channel involved in mechanosensation and hearing by the chordotonal neurons in *Drosophila*^38^. We predicted that constitutive activation of the channel by NAM would result in abrogation of the ability of Nan–Iav to mediate behavioural responses or perhaps result in death of the chordotonal neurons. Consistent with our prediction, treatment of wild-type third instar *Drosophila* larvae with NAM, but not NA, diminishes their response to a vibrational (sound) stimulus (Fig. 7a), similar to loss of either *nan* or *iav* function^38^. To determine if NAM causes death of the channel-expressing cells, as in *C. elegans*, we visualized the chordotonal neurons via expression of GCaMP6, but we detected no sign of a cell death response (for example,Supplementary Movie 1), even after 30 min of treatment with 1 M NAM (*n*=6).

Because the chordotonal neurons failed to die, we were able to examine the effect of NAM on their signalling responses using GCaMP6 fluorescence. We first established that application of NAM to the animals elicits a signalling response; GCaMP6 fluorescence increases in the chordotonal neurons upon application of NAM, indicating an increase in cytosolic calcium (Fig. 7b,c,e and Supplementary Movie 1). We next tested if NAM influences the ability of the chordotonal neurons to respond directly to stimuli. We established that a mechanical (vibrational) stimulus to 3rd instar larvae results in a rapid and robust calcium influx in the chordotonals, as revealed by a transient increase in GCaMP6 fluorescence (Fig. 7b–e and Supplementary Movie 1). However, application of NAM to the cells effectively prevents any subsequent response to any mechanical stimulus (Fig. 7b,c,e and Supplementary Movie 1).

**Figure 8:**
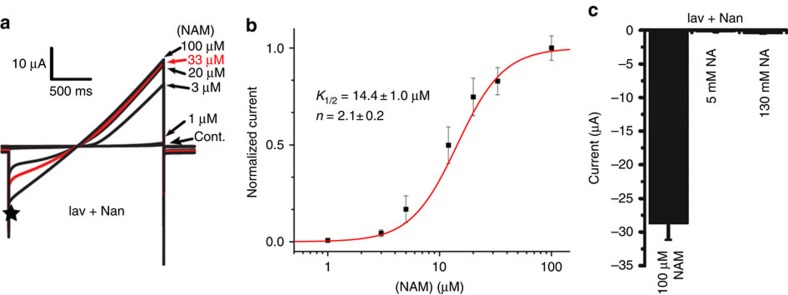
*Drosophila* Inactive (IAV) and Nanchung (NAN) channel is activated by NAM. (**a**,**b**) An example trace of the effect of NAM on the Nan–Iav channel. Large NAM-activated currents were observed in oocytes co-expressing Nan and Iav. Currents were recorded in response to 2 s voltage ramps from −100 to +100 mV from a −20 mV hold. Current size was measured at −100 mV (star), and the half maximal concentration (*K*_1/2_) for current activation by NAM was determined from the Hill plot shown in **b**. Points and bars in **b** represent mean±s.e.m. normalized current at a given concentration of NAM (*n*=5–13 oocytes per NAM concentration). The Hill coefficient (*n*) is estimated to be about 2.1. This is consistent with the estimated Hill coefficient for the OSM-9-OCR-4 channel (4d), suggesting that the number of NAM binding sites is conserved in invertebrate TRPV channels. *K*_1/2_ appears to be about 14 μM, which implies 4–5 fold greater affinity of NAM by the Nan–Iav channel compared with the OSM-9-OCR-4 channel (4d). The number of NAM binding sites for the Nan–Iav channel is estimated to be 2, which is consistent with the number of NAM binding sites for the OSM-9-OCR-4 channel. (**c**) Current is not induced in Nan–Iav channel by 5 mM NA (−0.188±0.020 μA, *n*=3); There is a slight increase in the conduction of the Nan–Iav channel upon exposure to 130 mM NA (−0.422±0.040 μA, *n*=4). Though this is a statistically significant increase (*P*<0.01, two-tailed *t*-test), this high level of NA (130 mM) elicited over 50-fold less current compared with 1,000-fold less NAM.

Finally, we also examined the activity of NAM on the Nan–Iav channel directly using the heterologous *Xenopus* system (Fig. 8). As with co-expression of OCR-4 and OSM-9, we detect a large current in response to NAM in oocytes co-expressing Nan and Iav (Fig. 8a,b). The *K*_1/2_ value for NAM, derived from a Hill fit of current size, is 14.4±1.0 μM (Fig. 8b). Again, the agonist activity of NAM is not shared by the related metabolite NA (Fig. 8c), and neither Nan (*n*=6 injections) nor Iav (*n*=5) alone produced active channels. We conclude that agonist activity of NAM on the invertebrate TRPV channel is evolutionarily conserved.

## Discussion

We have demonstrated that NAM, which is a form of vitamin B_3_ and an endogenous cellular metabolite, is an agonist of a heteromeric *C. elegans* TRPV channel that consists of OCR-4 and OSM-9 subunits. Moreover, agonist activity is evolutionarily conserved as NAM also activates the orthologous *Drosophila* Nan–Iav channel. We propose that activation of the OSM-9–OCR-4 channels is the trigger mechanism for NAM-induced uv1 and OLQ cell death in *C. elegans*. When NAM levels rise upon loss of the PNC-1 nicotinamidase that metabolizes NAM, the OCR-4 and OSM-9-expressing OLQ and uv1 cells likely undergo excitotoxic death. Aberrant activation of TRPV channels in humans has also been associated with cell death^39^,^40^, and a gain-of-function mutation in the *C. elegans* TRPN protein TRP-4 causes excitatory cell death of dopaminergic neurons^41^.

While OLQ and uv1 cells both die in response to NAM, it is unclear why the OLQ response is less robust than that of uv1. A difference in level of channel expression or access of NAM to the endogenous channels could explain these results. Similarly, the multiple OCR proteins that are co-expressed with *ocr-4* in uv1 cells could influence the response. However, we conclude from our studies that the insult is the same in both cell types but the response is likely influenced by cell-type specific factors. Indeed, although the OLQ and uv1 cells die rapidly in response to elevated OSM-9–OCR-4 channel agonist, a rapid cell death is not the only predicted response to elevated channel agonist levels. Instead, death in response to an acute ion imbalance is expected to be cell-type dependent. Consistent with this prediction, function of some TRPV channel-expressing cells is disrupted by NAM without a death response. Most clearly in the *Drosophila* chordotonal neurons, application of the agonist prevents the chordotonal neurons from responding to further stimuli without death of the neurons.

Sustained presence of agonist is predicted to interfere with functions of cells that express the TRPV channel in two ways. First, the constitutive opening and likely desensitization of the channel would interfere with any signalling event and behavioural output for which the channel is required. In this scenario prolonged presence of agonist would mimic the genetic loss of the TRPV channel. We found evidence of this type of effect of NAM in the *Drosophila* sound-response assay and the *C. elegans* nose-touch phenotypes.

Second, constitutive opening and likely desensitization of the TRPV channel might be expected to disrupt the physiology of the cell, and perhaps even kill the cell, via ion imbalance or other feedback mechanisms, thereby disrupting other cellular processes in which the TRPV channel has no direct role. In this scenario, the effects of the agonist might actually be blocked by loss of the channel, protecting other cellular functions. Again, we found evidence of this effect in the Egl and the foraging phenotypes in *C. elegans* where disruption of egg laying muscle function and the death of the OLQ cells results in phenotypes that are not TRPV dependent *per se*.

It has proven difficult to establish channel activity for the *C. elegans* TRPV channels in a heterologous system^13^,^16^,^18^. However, our results suggest that there is no strict requirement for other non-TRPV subunits for trafficking or activity as had been suggested. The soluble agonist NAM reveals that appropriate combinations of *C. elegans* TRPV channel subunits form functional channels in a heterologous system. Our results also provide insight into NAM interaction with the channel. The maximum value of a Hill coefficient is predictive of the number of binding sites for a ligand on its receptor; therefore it is likely that there are at least two NAM binding sites on the OSM-9–OCR-4 channel. Combined with our stoichiometry experiments, these data suggest that NAM might bind to only one of the subunits or to the interface of two subunits. The development of this heterologous system will provide an important tool for further structure/function studies of *C. elegans* TRPV channels to answer this and other structural questions. We have already used this discovery to show for the first time that functional OSM-9–OCR-4 channels most likely have two of each subunit in the active channel.

Does NAM affect other TRPV channels? Our results suggest that it might. First, the effect of NAM is conserved between the *Drosophila* TRPV channel and at least one *C. elegans* TRPV channel. The presence of multiple OCR paralogs in *C. elegans* raises the question of whether all heteromeric combinations of *C. elegans* TRPV channels will respond to NAM. We failed to detect channel activity with or without NAM upon co-expression of OCR-2 and OSM-9, but did not yet test other OSM-9–OCR combinations. The nose touch experiments suggest some sensitivity of OSM-9 perhaps in combination with another subunit to NAM. Finally, the egg-laying behaviour experiments suggest intriguing differences in how this phenotype responds to mutation of *osm-9* versus *ocr-4*, suggesting possible involvement of TRPV channels with alternate OCR subunits in this NAM-induced phenotype as well as function of OSM-9–OCR-4 in a previously undetected location. Nonetheless, it is clear that not all *osm-9* mutant phenotypes are mimicked by NAM accumulation; we examined *pnc-1* mutants but found no indication of an osmo-sensation phenotype. Finally, the 2:2 stoichiometry we detected provides for the possibility that up to two different OCR subunits could co-exist in the same channel. Thus, there are clearly intriguing questions to be addressed regarding the function and NAM sensitivity of the multiple OCR paralogs in *C. elegans*.

Both NA and NAM are forms of vitamin B_3_ used for biosynthesis of NAD^+^. Our results revealed that NA is not an agonist of the OSM-9–OCR-4 or Nan–Iav channel. However, the NA form of vitamin B_3_ is an apparent low-affinity agonist of mammalian TRPV channels^42^,^43^. Our identification of another form of vitamin B_3_ as a potent TRPV agonist raises intriguing possibilities for metabolic regulation of channel activity. Identification of a soluble agonist for a *C. elegans* TRPV combined with recent identification of an insecticide as a *Drosophila* channel agonist^44^ also paves the way for closer examination of the evolution of TRPV activation mechanisms.

## Methods

### *C. elegans* culture and strains

Strains were grown under standard conditions with OP50 *Escherichia coli* as a food source at 20 °C (ref. 45).

Strains used: BL5715 *inIs179*[*ida-1*::GFP] II (ref. 46); HV560 *inIs179*[*ida-1*::GFP] II*; pnc-1*(*pk9605*) IV—this is a null allele^22^; CX10 *osm-9(ky10)* IV (ref. 13); LX950 *ocr-4(vs137)* IV (ref. 18); HV720 *unc-119(ed3)* III; *psEx279* (see below, this strain has GFP expression in OLQ and uv1 cells only); HV695 *pnc-1*(*pk9605*) IV; *psEx279*[*ocr-4p*::GFP]; HV784 *inIs179*[*ida-1*::GFP] II; *pnc-1(pk9605) ocr-4(vs137)* IV; HV832 *inIs179*[*ida-1*::GFP] II; *pnc-1(pk9605) osm-9(ky10)* IV; CX4533 *ocr-1(ok132)* V (ref. 14); LX671 *ocr-2(vs29)* IV (ref. 18); VM396 *ocr-2(ak47)* IV (ref. 14); RB1734 *ocr-3(ok1559)* X (ref. 47).

### *psEx279* transgene construction

We created an *ocr-4p::gfp* transcriptional fusion to label the uv1 cells for other purposes and used it in this study as an OLQ marker. We amplified 1,015 bp upstream of the start site of *ocr-4* (*ocr-4* F1 5′ GCATGCCACTCAACAACCCATTTGC; *ocr-4* R1 5′ GGATCCTAATACAAGTTAGATTCAGAGAATATTTTACT) and ligated the product to pPD95.69 (Addgene plasmid 1491) using Sph I and Bam HI. We subsequently amplified 570 bp of the *ocr-4* 3′ UTR (*ocr-4* F2 5′ GAATTCTTTTTTTTACTGTTTCATTCTCTTCCTAAA; *ocr-4* R2 5′ ACTAGTTTGATAAGATAACATTCCACTCGTTAG) and ligated to the construct above using Eco RI and Spe I. After sequence confirmation, 100 ng μl^−1^ plasmid was injected into *unc-119(ed3)* with 60 ng μl^−1^ of *unc-119*(+) DNA (ref. 48). The resulting transgene is *psEx279*.

### Construction of GFP fusions plasmids

GFP-OCR-4 and GFP-Kv2.1 expression constructs were made by fusing eGFP with a short peptide linker (QQQGQQA or QQQAST, respectively) to the full channel ORF using overlap PCR, and constructs were sequence verified.

### uv1 and OLQ cell death phenotypic analysis

uv1 and QLQ cell death was analysed by fluorescence microscopy (Fig. 1e) or differential interference contrast (DIC) microscopy (Fig. 2a). In fluorescence microscopy assays *inIs179*[*ida-1*::GFP] (ref. 46) and *psEx279*[*ocr-4p*::GFP] mark the four uterine uv1 cells and the four OLQ cells, respectively. We scored uv1 and OLQ cell death by examining late L4 (uv1) and late L3 (OLQ) animals for the presence of living (no sign of degeneration) GFP+ cells. Data is reported as per cent of dead cells={(*n* × 4)−cells alive} × 100/(*n* × 4), where *n* is the total number of animals scored. In DIC microscopy assays, late L3 (OLQ) or L4 animals (uv1) were mounted in a drop of 1 M NAM on a 2% agarose pad on a slide and incubated at least two minutes. Animals were then counted for presence or absence of cell corpses over twenty minutes. Data is reported as per cent of animals with dead cells=(animals with at least one cell corpse present/*n*) × 100, where *n* is the total number of animals scored.

### Time course experiments

One wild-type animal was mounted on a slide with 2% agarose in M9 medium and topped with a cover slip. The animal was observed for the correct stage (L4 and late L3 animals for uv1 and OLQ death, respectively) and healthy cells pre-treatment. The cover slip was then lifted, a drop of 1 M NAM was added (time 0) and the cover slip was replaced. Time was recorded when the first evidence of cell death (nuclear disintegration) was observed. If no cell death was observed for 20 min, the assay was stopped.

### Egg laying assay

Each L4 stage hermaphrodite was raised individually and transferred serially to a new plate daily to avoid crowding with progeny. The animal was monitored for egg laying daily for 3–4 days. If the embryos hatched inside the mother's body instead of being expelled through the vulva, the animal was scored as Egl. Data are presented as the percentage of Egl animals.

### Nose-touch assay

We prepared assay plates by placing a small drop of an overnight OP50 culture on an nematode growth medium (NGM) plate and storing plates at 4 °C for up to 10 days. Plates were moved to room temperature for an hour before use. We placed a single worm on a plate and allowed it to move around for at least 5 min. Then we brought a thin eyebrow hair into the path of a forward moving animal to cause it to collide nose on. Either halting of forward locomotion or initiation of backward movement was scored as a positive response. Each animal was touched 10 times with 10–60 s intervals between the touches. Experimenters were blind to the genotype or condition. Data are presented as the percentage of all touches that produced a positive response.

### Foraging

Assay plates were identical to nose touch assays. Animals were placed on the plate, allowed to acclimate for at least 1 min, and then observed at 50 × magnification during spontaneous forward movement for 1 min. Typically, as the animal moves forward, the tip of the nose protrudes towards the front while making small exploratory movements to the side, keeping its nose tip straight. Animals however occasionally curve their heads while foraging as if they were looking sideways while moving forward, and this phenotype is observed more frequently in some foraging mutants^31^ (Fig. 6a). Data are presented as the percentage of all observed animals per genotype or condition that had one or more observed exaggerated head bends. Observers were blind to the genotype or condition for all assays.

### *Drosophila* tone response

Third instar *Drosophila* larvae were pretreated with 1 M NAM or 1 M NA for ten minutes by soaking. After treatment, larvae were transferred to an NGM plate (no *E. coli*), placed on a speaker (Big Jambox by Jawbone) and exposed to a 500 Hz tone at 90 decibels (http://soundbible.com/1396-500-Hz-Tone.html). Retraction of the mouth was recorded as a positive startle response^49^. Each larva was tested five times with a 2–3 s recovery between tones to ensure that larvae were moving forward when scoring occurred. Only forward moving larvae were scored. Data are presented as the percentage of all observed stimuli that resulted in a startle response. Observers were blind to the treatment condition.

### *Xenopus* oocyte expression and electrophysiology

cRNAs were made from *osm-9* cDNA (in vector pGEMHE, gift of Dr C. Bargmann, Rockefeller University), *ocr-4* cDNA (gift of Dr Y. Kohara, National Institute of Genetics, Japan, transferred to pOX (ref. 50), *iav* cDNA (ref. 38) (gift of J. Kim lab, KRIBB, South Korea transferred into pCR4-TOPO), and *nan* cDNA (synthesized and purchased from GenScript, Piscataway, NJ, in pcDNA3.1) using mMessage Machine kits (Ambion, TX) and injected at ∼50 ng per oocyte. Oocyte preparation, injection and culture have been described^49^,^50^. cRNAs were injected individually and in the following combinations: *osm-9*+*ocr-4*, *iav*+*nan*. Recordings were made one to three days after injection at room temperature using standard two electrode voltage clamp techniques^52^ in a solution containing (in mM): 98 Na^+^, 2 K^+^, 1 Mg^2+^, 1 Ca^2+^, 104 Cl^−^, 5 HEPES (pH 7.5 with NaOH). All recordings were performed using a Dagan CA 1-B oocyte clamp (Dagan, MN) and Clampex (Molecular Devices, CA). Currents were analysed offline using Clampfit (Molecular Devices, CA). Statistics and curve fitting were performed using Origin (OriginLab, MA).

### Hold protocol recordings

Eggs were held at −80 mV for the duration of the recording. Following 30 s of exposure to the bath solution, eggs were exposed to bath plus 10 μM NAM for 90 s, immediately followed by exposure to bath plus 100 μM NAM for 40 s. 100 μM NAM was then washed out by bath solution.

### Ramp protocol recordings

Eggs were held at −20 mV for 50 ms. Then the voltage was changed to −100 mV and continuously increased to +100 mV over a period of 2 s. NAM at varying concentrations was added until the amount of current at −100 mV reached steady state. The time in between sweeps was 7.5 s.

### Total internal reflection microscopy

TIRF microscopy was performed on a Nikon TE-2000 inverted microscope outfitted with a 60 × N.A. 1.45 objective (Nikon). An 80 mW argon ion laser (Spectra Physics) was used for illumination, and an iXon Ultra 888 EMCCD (Andor Technology) camera was used for detection. Movies were acquired at five frames per second using Micro-Mananger software (http://www.micro-manager.org). Background-corrected fluorescence intensity signals for single channels were acquired from raw movies using a custom-built, semi-automated analysis tool coded in MATLAB (Mathworks), which summed counts in a 7 pixel diameter circle drawn around candidate channels, and photobleaching steps were determined using a step-finding algorithm^51^. Bleach event detection efficiency was calculated from a least squares fit of the step count assuming four GFPs per channel and a binomial distribution of missed events. OSM-9–OCR-4 stoichiometry was reverse-calculated from the aggregate step counts in the same manner using the Kv2.1 detection efficiency value (0.69), a value consistent with other published studies^27^,^29^,^30^.

Only stationary spots were analysed. Before TIRF, vitelline envelopes were removed to expose the oocyte membrane^52^, allowing for direct contact with the optical glass dish. Oocytes were placed in hyperosmotic stripping solution (98 mM NaCl, 2 mM KCl, 1 mM each of MgCl_2_ and CaCl_2_, 5 mM HEPES, 100 units ml^−1^ Pen-Strep, 2.5 mM Na-pyruvate, pH 7.2 with NaOH, 200 mM sucrose) to shrink the oocyte, and then fine forceps were used to mechanically remove the spatially separated vitelline envelope.

### *In vivo* analysis of *Drosophila* chordotonal neurons

To visualize the activity of chordotonal neurons, flies expressing Gal4 under the pan-neuronal promoter ELAV and UAS driven mRFP1 (ref. 53) were crossed to a fly line containing a UAS driven genetically encoded calcium sensor, GCaMP6 (medium kinetics)^54^. Third instar larva were rinsed in PBS and mounted laterally on an agar covered slide for viewing. Chordotonal neurons in lateral chordotonal organ 5 (LCh5) were selected for imaging. Z-stacks through the chordotonal organ were acquired with a Zeiss LSM510 confocal microscope using a 63 × oil N.A. 1.4 objective (Zeiss). Scan settings were 512 × 512, 12 bit, with a pixel time of 1.6 μs, which resulted in a scan rate of ∼1 frame per second. The pinhole was set at 872 to result in a 6 μm optical slice that was offset 3 μm for each of four slices. Each stack was acquired over 4 s, with 6 s gaps between stacks. Excitation and emission were performed with standard GFP settings with regard to laser, dichroic and band-pass filter. Stimulation was achieved by gently rubbing the base of the microscope with the ribbed bottom of a glass bottle. After two to three such paired recordings, we applied solutions of either 1 M NAM or 1 × PBS to the side of the coverslip, taking care not to touch the slide with the pipet. The solution was drawn to cover the larva via capillary action. After ∼20 s, another series of two pairs of resting and stimulation images were acquired.

Image analysis was performed with Fiji (ImageJ). Z-stacks for quantification were flattened with the ‘z-project' command, and quantified by using the polygon selection tool to tightly select the dendrites, cell bodies and proximal axons of the entire chordotonal neuron cluster (for example,Fig. 7b) before using the ‘measure' command. Measurements were exported to Excel for recording and statistical analysis.

A separate assay was performed for demonstration purposes (Supplementary Movie 1) with continuous scanning and paired rest and stimulation periods before and after the addition of 1 M NAM. This time-lapse image was imported into Fiji, labelled, and saved as an. AVI file at 4 fps. Fiji's ‘Fire' look up table was applied to all images and the movie for the purpose of better demonstrating the dynamic range of the neuronal response to physical and chemical stimulation.

### Statistical analysis

Statistical tests and sample sizes for each experiment are described in figure legends with relevant *P* values. The statistical tests were two-tailed. Statistical analyses were performed in Excel or GraphPad Software.

### Data availability

The authors declare that the data supporting the findings of this study are available within the article and its Supplementary Information Files or available from the corresponding author upon reasonable request.

## Additional information

**How to cite this article:** Upadhyay, A. *et al*. Nicotinamide is an endogenous agonist for a *C. elegans* TRPV OSM-9 and OCR-4 channel. *Nat. Commun.* **7,** 13135 doi: 10.1038/ncomms13135 (2016).

## Supplementary Material

Supplementary Information Supplementary Figures 1-4

Supplementary Movie 1 GCaMP6 fluorescence in chordotonal neurons. Continuous imaging of chordotonal neurons was performed to demonstrate behaviour in response to mechanical stimulation and NAM administration. Imaging begins with the neurons at rest. Alternating periods stimulation and rest were performed before administration of NAM, after which alternating periods of stimulation and rest were observed and labelled. Note the difference in fluorescence intensity upon stimulation in the absence of NAM, the increase in fluorescence upon NAM administration, and the lack of response to stimulation in the presence of NAM. Continuous imaging was performed at 20x magnification with a zoom factor of 5 on a Zeiss LSM 510 with LSM software 4.0 sp2.

## References

[b1] Tóth A. . Expression and distribution of vanilloid receptor 1 (TRPV1) in the adult rat brain. Mol. Brain Res. 135, 162–168 (2005). 15857679 10.1016/j.molbrainres.2004.12.003

[b2] Nilius B. & Owsianik G. The transient receptor potential family of ion channels. Genome Biol. 12, 218 (2011). 21401968 10.1186/gb-2011-12-3-218 PMC3129667

[b3] Venkatachalam K., Luo J. & Montell C. in Mamm. Transient Recept. Potential Cation Channels eds Nilius, B. & Flockerzi, V.937–962Springer International Publishing (2014).

[b4] Julius D. TRP channels and pain. Annu. Rev. Cell Dev. Biol. 29, 355–384 (2013). 24099085 10.1146/annurev-cellbio-101011-155833

[b5] Kahn-Kirby A. H. & Bargmann C. I. TRP channels in *C. elegans*. Annu. Rev. Physiol. 68, 719–736 (2006). 16460289 10.1146/annurev.physiol.68.040204.100715

[b6] Kahn-Kirby A. H. . Specific polyunsaturated fatty acids drive TRPV-dependent sensory signaling *in vivo*. Cell 119, 889–900 (2004). 15607983 10.1016/j.cell.2004.11.005

[b7] Zygmunt P. M. . Vanilloid receptors on sensory nerves mediate the vasodilator action of anandamide. Nature 400, 452–457 (1999). 10440374 10.1038/22761

[b8] Kedei N. . Analysis of the native quaternary structure of vanilloid receptor 1. J. Biol. Chem. 276, 28613–28619 (2001). 11358970 10.1074/jbc.M103272200

[b9] Kuzhikandathil E. V. . Functional analysis of capsaicin receptor (vanilloid receptor subtype 1) multimerization and agonist responsiveness using a dominant negative mutation. J. Neurosci. 21, 8697–8706 (2001). 11698581 10.1523/JNEUROSCI.21-22-08697.2001 PMC6762288

[b10] Hellwig N., Albrecht N., Harteneck C., Schultz G. & Schaefer M. Homo- and heteromeric assembly of TRPV channel subunits. J. Cell Sci. 118, 917–928 (2005). 15713749 10.1242/jcs.01675

[b11] Hoenderop J. G. J. . Homo- and heterotetrameric architecture of the epithelial Ca^2+^ channels TRPV5 and TRPV6. EMBO J. 22, 776–785 (2003). 12574114 10.1093/emboj/cdg080 PMC145440

[b12] Rutter A. R., Ma Q., Leveridge M. & Bonnert T. P. Heteromerization and colocalization of T and T rpV2 in mammalian cell lines and rat dorsal root ganglia. Neuroreport 16, 5–9 (2005). 16237318 10.1097/01.wnr.0000185958.03841.0f

[b13] Colbert H. A., Smith T. L. & Bargmann C. I. OSM-9, a novel protein with structural similarity to channels, is required for olfaction, mechanosensation, and olfactory adaptation in *Caenorhabditis elegans*. J. Neurosci. 17, 8259–8269 (1997). 9334401 10.1523/JNEUROSCI.17-21-08259.1997 PMC6573730

[b14] Tobin D. . Combinatorial expression of TRPV channel proteins defines their sensory functions and subcellular localization in *C. elegans* neurons. Neuron 35, 307–318 (2002). 12160748 10.1016/s0896-6273(02)00757-2

[b15] Goodman M. B. & Schwarz E. M. Transducing touch in *Caenorhabditis elegans*. Annu. Rev. Physiol. 65, 429–452 (2003). 12524464 10.1146/annurev.physiol.65.092101.142659

[b16] De Bono M., Tobin D. M., Davis M. W., Avery L. & Bargmann C. I. Social feeding in *Caenorhabditis elegans* is induced by neurons that detect aversive stimuli. Nature 419, 899–903 (2002). 12410303 10.1038/nature01169 PMC3955269

[b17] Zhang S., Sokolchik I., Blanco G. & Sze J. Y. *Caenorhabditis elegans* TRPV ion channel regulates 5HT biosynthesis in chemosensory neurons. Development 131, 1629–1638 (2004). 14998926 10.1242/dev.01047

[b18] Jose A. M., Bany I. A., Chase D. L. & Koelle M. R. A specific subset of transient receptor potential vanilloid-type channel subunits in *Caenorhabditis elegans* endocrine cells function as mixed heteromers to promote neurotransmitter release. Genetics 175, 93–105 (2007). 17057248 10.1534/genetics.106.065516 PMC1774992

[b19] Xiao R. & Xu X. Z. S. Function and regulation of TRP family channels in *C. elegans*. Pflugers Arch. 458, 851–860 (2009). 19421772 10.1007/s00424-009-0678-7 PMC2857680

[b20] Alkema M. J., Hunter-Ensor M., Ringstad N. & Horvitz H. R. Tyramine functions independently of octopamine in the *Caenorhabditis elegans* nervous system. Neuron 46, 247–260 (2005). 15848803 10.1016/j.neuron.2005.02.024

[b21] Wang W. . Comparative metabolomic profiling reveals that dysregulated glycolysis stemming from lack of salvage NAD+ biosynthesis impairs reproductive development in *Caenorhabditis elegans*. J. Biol. Chem. 290, 26163–26179 (2015). 26350462 10.1074/jbc.M115.662916 PMC4646267

[b22] Vrablik T. L., Huang L., Lange S. E. & Hanna-Rose W. Nicotinamidase modulation of NAD+ biosynthesis and nicotinamide levels separately affect reproductive development and cell survival in *C. elegans*. Development 136, 3637–3646 (2009). 19820182 10.1242/dev.028431 PMC2761111

[b23] Huang L. & Hanna-Rose W. EGF signaling overcomes a uterine cell death associated with temporal mis-coordination of organogenesis within the *C. elegans* egg-laying apparatus. Dev. Biol. 300, 599–611 (2006). 16963018 10.1016/j.ydbio.2006.08.024

[b24] Crook M., Upadhyay A. & Hanna-Rose W. Necrosis in *C. elegans*. Methods Mol. Biol. 1004, 171–182 (2013). 23733577 10.1007/978-1-62703-383-1_13 PMC4485986

[b25] Vlachos M. & Tavernarakis N. Non-apoptotic cell death in *Caenorhabditis elegans*. Dev. Dyn. 239, 1337–1351 (2010). 20108319 10.1002/dvdy.22230

[b26] Lindy A. S. . TRPV channel-mediated calcium transients in nociceptor neurons are dispensable for avoidance behaviour. Nat. Commun. 5, 4734 (2014). 25178952 10.1038/ncomms5734 PMC4164786

[b27] Ulbrich M. H. & Isacoff E. Y. Subunit counting in membrane-bound proteins. Nat. Methods 4, 319–321 (2007). 17369835 10.1038/NMETH1024 PMC2744285

[b28] Nakajo K., Ulbrich M. H., Kubo Y. & Isacoff E. Y. Stoichiometry of the KCNQ1–KCNE1 ion channel complex. Proc. Natl Acad. Sci. 107, 18862–18867 (2010). 20962273 10.1073/pnas.1010354107 PMC2973890

[b29] Kitazawa M., Kubo Y. & Nakajo K. The stoichiometry and biophysical properties of the Kv4 potassium channel complex with K+ channel-interacting protein (KChIP) subunits are variable, depending on the relative expression level. J. Biol. Chem. 289, 17597–17609 (2014). 24811166 10.1074/jbc.M114.563452 PMC4067195

[b30] Yu Y. . Structural and molecular basis of the assembly of the TRPP2/PKD1 complex. Proc. Natl Acad. Sci. USA 106, 11558–11563 (2009). 19556541 10.1073/pnas.0903684106 PMC2710685

[b31] Kindt K. S. . *Caenorhabditis elegans* TRPA-1 functions in mechanosensation. Nat. Neurosci. 10, 568–577 (2007). 17450139 10.1038/nn1886

[b32] Hart A. C., Sims S. & Kaplan J. M. Synaptic code for sensory modalities revealed by *C. elegans* GLR-1 glutamate receptor. Nature 378, 82–85 (1995). 7477294 10.1038/378082a0

[b33] Chatzigeorgiou M. & Schafer W. R. Lateral facilitation between primary mechanosensory neurons controls nose touch perception in *C. elegans*. Neuron 70, 299–309 (2011). 21521615 10.1016/j.neuron.2011.02.046 PMC3145979

[b34] Johnson R. W., Liu L. Y., Hanna-Rose W. & Chamberlin H. M. The *Caenorhabditis elegans* heterochronic gene lin-14 coordinates temporal progression and maturation in the egg-laying system. Dev. Dyn. 238, 394–404 (2009). 19161245 10.1002/dvdy.21837

[b35] Vrablik T. L., Wang W., Upadhyay A. & Hanna-Rose W. Muscle type-specific responses to NAD+ salvage biosynthesis promote muscle function in *Caenorhabditis elegans*. Dev. Biol. 349, 387–394 (2011). 21092737 10.1016/j.ydbio.2010.11.014 PMC3019288

[b36] Kaplan J. M. & Horvitz H. R. A dual mechanosensory and chemosensory neuron in *Caenorhabditis elegans*. Proc. Natl Acad. Sci. USA 90, 2227–2231 (1993). 8460126 10.1073/pnas.90.6.2227 PMC46059

[b37] Matsuura H., Sokabe T., Kohno K., Tominaga M. & Kadowaki T. Evolutionary conservation and changes in insect TRP channels. BMC Evol. Biol. 9, 228 (2009). 19740447 10.1186/1471-2148-9-228 PMC2753570

[b38] Gong Z. . Two interdependent TRPV channel subunits, inactive and Nanchung, mediate hearing in *Drosophila*. J. Neurosci. 24, 9059–9066 (2004). 15483124 10.1523/JNEUROSCI.1645-04.2004 PMC6730075

[b39] Reilly C. A. . Capsaicinoids cause inflammation and epithelial cell death through activation of vanilloid receptors. Toxicol. Sci. 73, 170–181 (2003). 12721390 10.1093/toxsci/kfg044 PMC2423488

[b40] Ryskamp D. A. . The polymodal ion channel transient receptor potential vanilloid 4 modulates calcium flux, spiking rate, and apoptosis of mouse retinal ganglion cells. J. Neurosci. 31, 7089–7101 (2011). 21562271 10.1523/JNEUROSCI.0359-11.2011 PMC3109951

[b41] Nagarajan A. . Progressive degeneration of dopaminergic neurons through TRP channel-induced cell death. J. Neurosci. 34, 5738–5746 (2014). 24760834 10.1523/JNEUROSCI.4540-13.2014 PMC3996206

[b42] Ma L., Lee B. H., Clifton H., Schaefer S. & Zheng J. Nicotinic acid is a common regulator of heat-sensing TRPV1-4 ion channels. Sci. Rep. 5, 8906 (2015). 25752528 10.1038/srep08906 PMC4894441

[b43] Ma L. . Nicotinic acid activates the capsaicin receptor TRPV1: potential mechanism for cutaneous flushing. Arterioscler. Thromb. Vasc. Biol. 34, 1272–1280 (2014). 24675661 10.1161/ATVBAHA.113.303346 PMC4063526

[b44] Nesterov A. . TRP channels in Insect stretch receptors as insecticide targets. Neuron 86, 665–671 (2015). 25950634 10.1016/j.neuron.2015.04.001

[b45] Brenner S. The genetics of *Caenorhabditis elegans*. Genetics 77, 71–94 (1974). 4366476 10.1093/genetics/77.1.71 PMC1213120

[b46] Zahn T. R., Macmorris M. A., Dong W., Day R. & Hutton J. C. IDA-1, a *Caenorhabditis elegans* homolog of the diabetic autoantigens IA-2 and phogrin, is expressed in peptidergic neurons in the worm. J. Comp. Neurol. 429, 127–143 (2001). 11086294 10.1002/1096-9861(20000101)429:1<127::aid-cne10>3.0.co;2-h

[b47] C. elegans Deletion Mutant Consortium. large-scale screening for targeted knockouts in the *Caenorhabditis elegans* genome. G3 (Bethesda) 2, 1415–1425 (2012). 23173093 10.1534/g3.112.003830 PMC3484672

[b48] Maduro M. & Pilgrim D. Identification and cloning of unc-119, a gene expressed in the *Caenorhabditis elegans* nervous system. Genetics 141, 977–988 (1995). 8582641 10.1093/genetics/141.3.977 PMC1206859

[b49] Zhang W., Yan Z., Jan L. Y. & Jan Y. N. Sound response mediated by the TRP channels NOMPC, NANCHUNG, and INACTIVE in chordotonal organs of *Drosophila* larvae. Proc. Natl Acad. Sci. USA 110, 13612–13617 (2013). 23898199 10.1073/pnas.1312477110 PMC3746866

[b50] Jegla T. & Salkoff L. A novel subunit for shal K+ channels radically alters activation and inactivation. J. Neurosci. 17, 32–44 (1997). 8987734 10.1523/JNEUROSCI.17-01-00032.1997 PMC6793676

[b51] Chen Y., Deffenbaugh N. C., Anderson C. T. & Hancock W. O. Molecular counting by photobleaching in protein complexes with many subunits: best practices and application to the cellulose synthesis complex. Mol. Biol. Cell 25, 3630–3642 (2014). 25232006 10.1091/mbc.E14-06-1146 PMC4230622

[b52] Jegla T. . Expanded functional diversity of shaker K(+) channels in cnidarians is driven by gene expansion. PLoS ONE 7, e51366 (2012). 23251506 10.1371/journal.pone.0051366 PMC3519636

[b53] Campbell R. E. . A monomeric red fluorescent protein. Proc. Natl Acad. Sci. USA 99, 7877–7882 (2002). 12060735 10.1073/pnas.082243699 PMC122988

[b54] Chen T.-W. . Ultrasensitive fluorescent proteins for imaging neuronal activity. Nature 499, 295–300 (2013). 23868258 10.1038/nature12354 PMC3777791

