# Supplementary material for: Nicotinamide is an endogenous agonist for a C. elegans TRPV OSM-9 and OCR-4 channel
Source: Nat Commun. 2016 Oct 12;7:13135. doi: 10.1038/ncomms13135 (PMC5064019; doi:10.1038/ncomms13135)
Supplement: Supplementary Information — Supplementary Figures 1-4 [file ncomms13135-s1.pdf]

Supplementary Figure 1

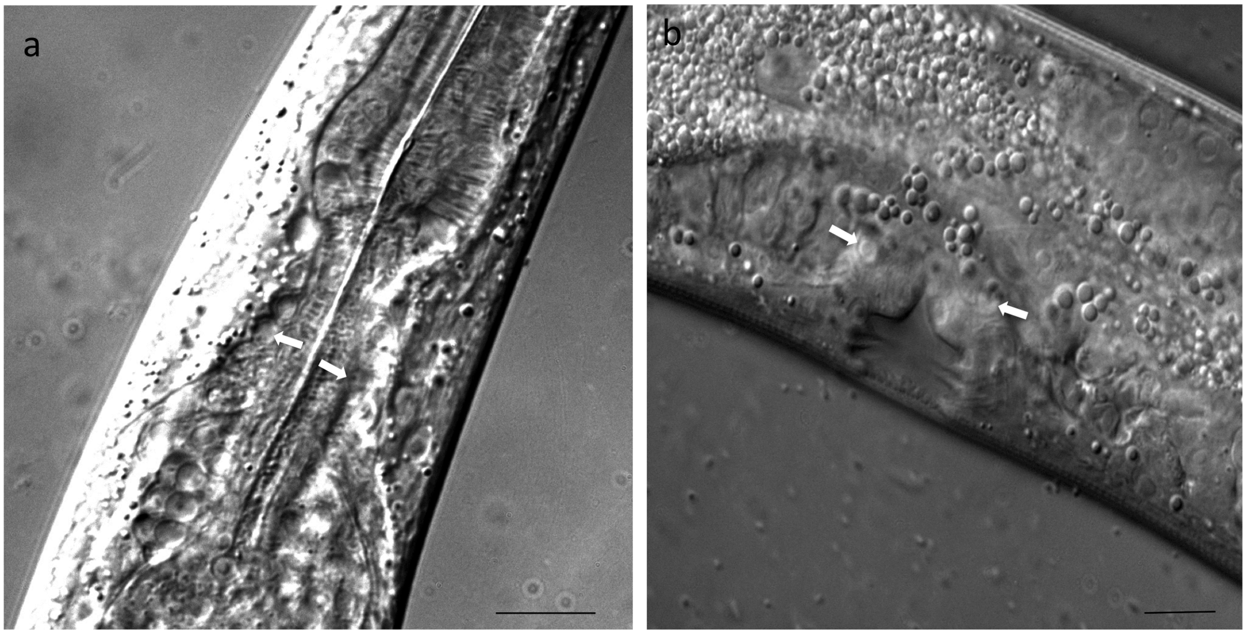

Supplementary Figure 1

Healthy OLQ and uv1 cells are of similar size to neighboring cells. a) White arrows point to the normal OLQ cell body in a wild-type animal carrying the *ocr-4p*::GFP transgene. b) White arrows point to normal uv1 cells. Scale bars are 10 μm.

Supplementary Figure 2

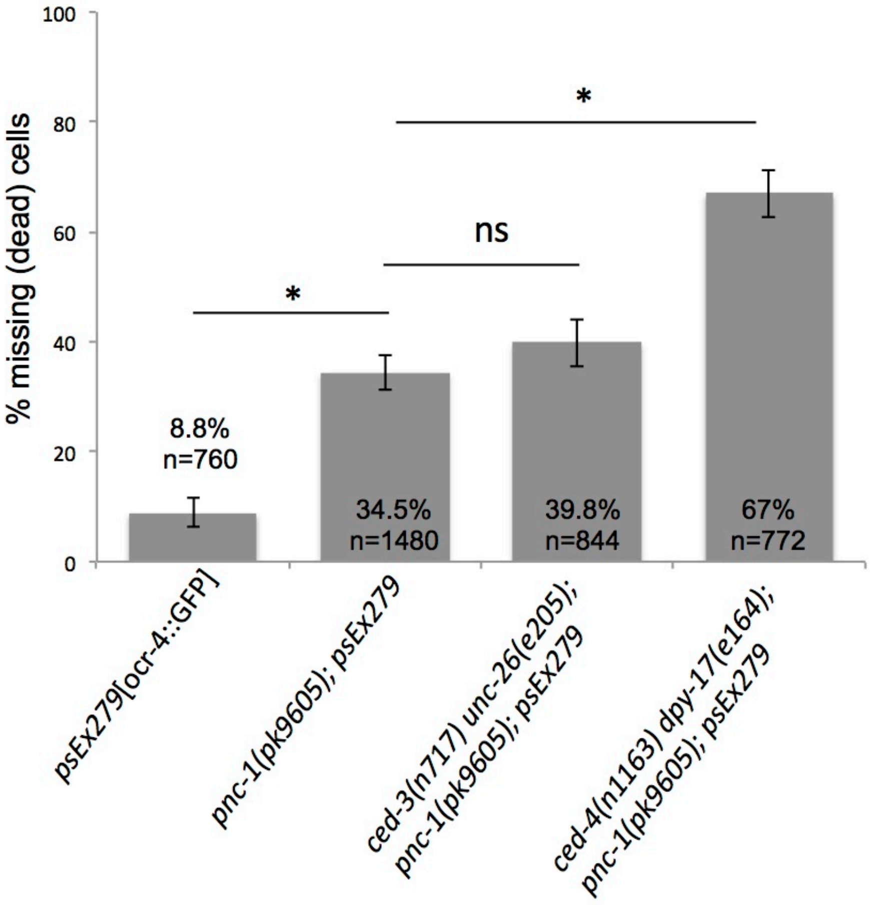

Supplementary Figure 2. Loss of *ced-3* and *ced-4* function does not prevent OLQ cell death in *pnc-1.*

The percentage of OLQ cells that are missing in adulthood (dead) for each genotype is reported. Actual percentages and sample sizes (# of cells examined) are indicated on each bar. Error bars represent 99% confidence intervals. *p <0.001, calculated using Fisher's exact test.

Supplementary Figure 3

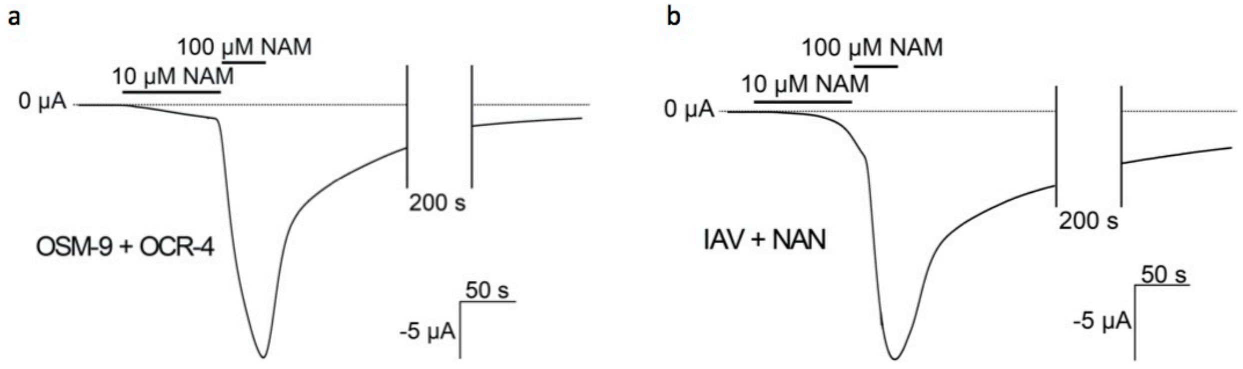

Supplementary Figure 3. Current traces of invertebrate TRPV channels in response to NAM.

Xenopus oocytes expressing a) OSM-9 and OCR-4 or b) Iav and Nan showed slowly activating inward currents following application of 10 µM NAM, then large, rapid inward currents following exposure to 100 µM NAM. Peak inward current before washout was approximately -23 µA for OSM-9/OCR-4 and approximately -17 µA for Iav/Nan. Washout of NAM appeared to occur in a biphasic manner, although it is unclear if the late slow current during washout is due to lingering NAM effect on the channels or due to activation of native currents.

Supplementary Figure 4

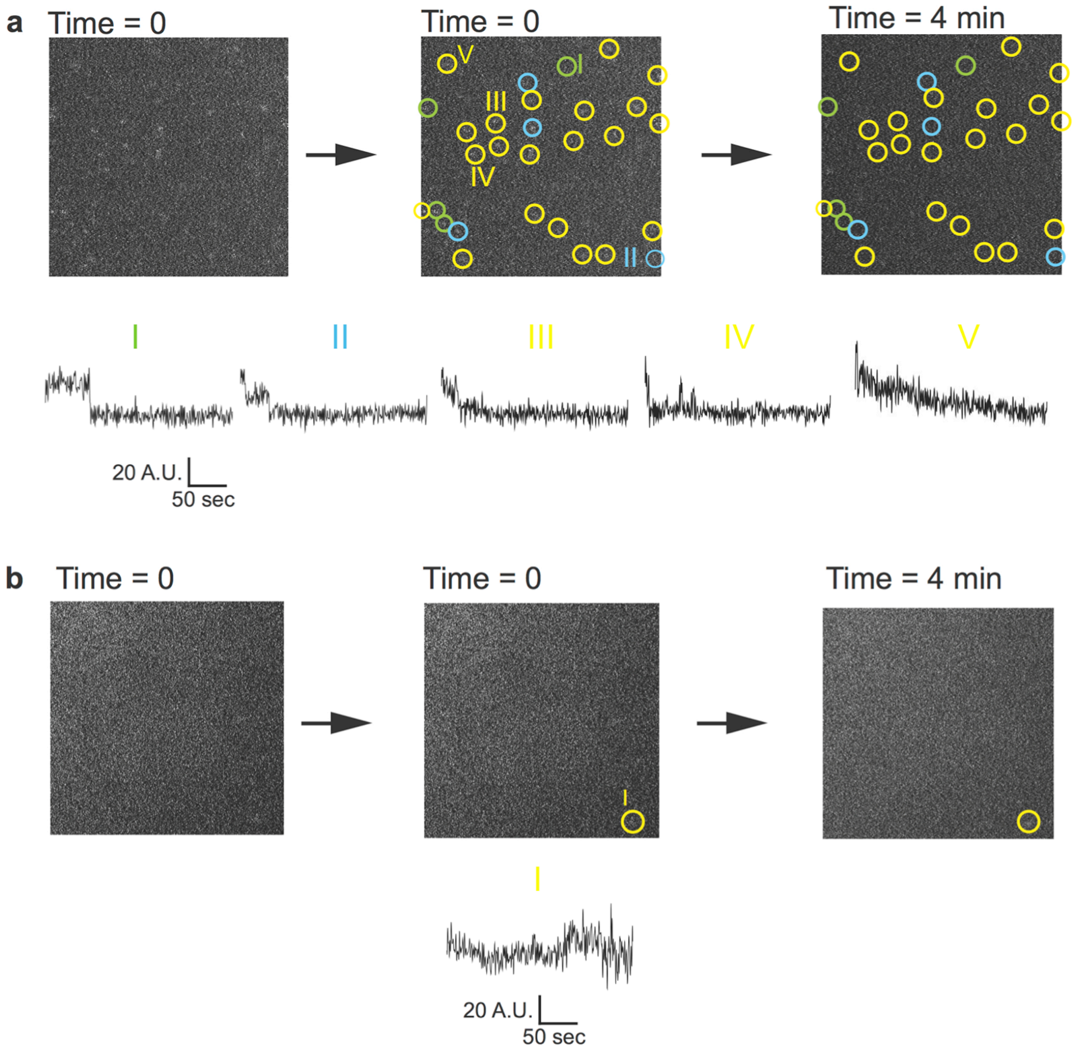

Supplementary Figure 4. Sample images and example analyses from TIRF photobleaching assay for OCR-4::GFP.

a) Oocytes injected with OCR-4::GFP and OSM-9 displayed many fluorescent spots on the membrane at the beginning of the observation period (top panel, time 0, representative frame of 300 square pixels from movie). Valid GFP-labeled channels are expected to photobleach in discrete steps. Moreover, photobleaching is permanent and fluorescence from a valid channel spot, which was photobleached, should not reappear. Thus, spots that failed to bleach (none in this field of view), lost fluorescence in a non-

stepwise fashion (V, outlined in yellow, with corresponding trace below), even if followed by a distinct bleaching event (III), or behaved erratically by “blinking” or losing and regaining fluorescence (IV) were excluded from the analysis because they either were not channels or represented events in which photobleaching steps could not be reliably counted. Every spot that was excluded from the analysis is indicated with a yellow outline. Every spot that was included as a final data point is indicated by a blue or green outline. These spots photobleached in one (I, outlined in green with corresponding trace below) or two-step events (II). In contrast, when we ran similar experiments on homotetrameric channels formed by GFP::Kv2.1, spots bleaching in 1, 2, 3, or 4 discrete steps were observed (Fig. 5a). Most spots were no longer visible at the end of the observation period (bottom panel, time 4 min).

b) In contrast to the oocytes injected with both subunits, there are few fluorescent spots on the membrane at the beginning of the observation period (top panel, time 0) in oocytes injected with OCR-4::GFP alone. Those present either failed to photobleach (none in this field of view) or fluoresced erratically over the course of the observation (I, outlined in yellow with corresponding trace below) suggesting that they were not GFP-labeled channels.
